# Muscle Synergies in Patients With Multiple Sclerosis Reveal Demand-Specific Alterations in the Modular Organization of Locomotion

**DOI:** 10.3389/fnhum.2020.593365

**Published:** 2021-01-27

**Authors:** Lars Janshen, Alessandro Santuz, Adamantios Arampatzis

**Affiliations:** ^1^Department of Training and Movement Sciences, Humboldt-Universität zu Berlin, Berlin, Germany; ^2^Berlin School of Movement Science, Humboldt-Universität zu Berlin, Berlin, Germany

**Keywords:** muscle synergies, multiple sclerosis, locomotion, motor control, inclined walking, demand-specific

## Abstract

For patients with multiple sclerosis (MS), deficits in gait significantly reduce the quality of life. Using the concept of muscle synergies, this study investigated the modular organization of motor control during level and inclined walking in MS patients (MSP) compared with healthy participants (HP) to identify the potential demand-specific adjustments in motor control in MSP. We hypothesized a widening of the time-dependent activation patterns (motor primitives) in MSP to increase the overlap of temporally-adjacent muscle synergies, especially during inclined walking, as a strategy to increase the robustness of motor control, thus compensating pathology-related deficits. We analyzed temporal gait parameters and muscle synergies from myoelectric signals of 13 ipsilateral leg muscles using non-negative matrix factorization. Compared with HP, MSP demonstrated a widening in the time-dependent coefficients (motor primitives), as well as altered relative muscle contribution (motor modules), in certain synergies during level and inclined walking. Moreover, inclined walking revealed a demand-specific adjustment in the modular organization in MSP, resulting in an extra synergy compared with HP. This further increased the overlap of temporally-adjacent muscle synergies to provide sufficient robustness in motor control to accomplish the more demanding motor task while coping with pathology-related motor deficits during walking.

## Introduction

The chronic degenerative neurological disease of multiple sclerosis (MS) is inflammatory-mediated and results in the demyelination of the central nervous system (CNS) (Bo et al., 2006; Popescu and Lucchinetti, 2012). The heterogeneous pathological and clinical presentation of MS typically includes deficits in the sensory (Fling et al., 2014), motor (Lambert et al., 2001; Thoumie et al., 2005; Kalron et al., 2011), and cognitive functions (Wingerchuk et al., 2001). About 75% of multiple sclerosis patients (MSP) experience clinically relevant walking disturbances (Hobart et al., 2001; Kasser and Jacobs, 2014). These include lower cadence and shorter step length resulting in a reduced walking speed (Benedetti et al., 1999; Cameron and Wagner, 2011; Comber et al., 2017). In the MS disability spectrum, gait is perceived as the most important bodily function (Heesen et al., 2008), and up to 85% of MSP report mobility impairments (LaRocca, 2011). Correspondingly, it is reported that more than 50% of MSP fall within an observation period of 6 months (Cattaneo et al., 2002) or 1 year (Finlayson and Peterson, 2010). Compared with healthy age-matched controls, females with MS have three times higher risk of falls (Cameron et al., 2011). More than 45% of falls in MSP have been attributed to external perturbations, such as slipping or tripping (Matsuda et al., 2011). In middle-aged MSP, persons with mobility impairments who do not use an assistive device have the highest risk of multiple falls (Coote et al., 2014).

Walking requires muscle coordination that controls the activation level and timing of multiple muscles (Bernstein, 1967; Winter and Yack, 1987). As a commonly accepted hypothesis, the CNS may manage to overcome the complexity of motor control by activating functionally related muscles in common patterns called muscle synergies (Bernstein, 1967; Bizzi et al., 2008). Earlier studies that investigated the modular organization of motor control during walking and running in the presence of perturbations showed that a common strategy of the human system is to increase control robustness (i.e., ability to cope with perturbations) (Rabinovich and Abarbanel, 1998; Santuz et al., 2018a) to ensure safe locomotion (Santuz et al., 2018a, 2020a). In MSP, the pathology-related degeneration of the neuromuscular system and the resulting deficits in the motor control efficiency can be considered as perturbations generated from internal, physiological sources (Van Hooren et al., 2018). Assuming a neural origin of muscle synergies, neural pathologies might alter the modular organization of the neuromuscular system. This could result in changes of the number of muscle synergies as reported for patients with cerebral palsy (Steele et al., 2015; Yu et al., 2019) or post-stroke (Clark et al., 2010; Allen and Neptune, 2012; Seamon et al., 2018) associated with alterations of the time-dependent synergy component (i.e., motor primitives) (Martino et al., 2015; Lencioni et al., 2016; Seamon et al., 2018), as well as in the time-invariant muscle weights (i.e., motor modules) (Gizzi et al., 2011). Previous studies comparing MSP with healthy participants (HP) during walking reported no inevitable reduction in the number of muscle synergies (Lencioni et al., 2016; Janshen et al., 2020).

We and others proposed that the widening of motor primitives helps to ensure robust control in the presence of external and internal perturbations (Martino et al., 2014, 2015; Cappellini et al., 2016; Santuz et al., 2018a, 2019). The widening of motor primitives might be a compensatory mechanism adopted by the CNS to cope with the postural instability of locomotion resulting from these disturbances (Martino et al., 2014, 2015; Santuz et al., 2018a, 2020a; Janshen et al., 2020). A widening of the motor primitives in healthy adults has been reported during walking on an uneven surface (Santuz et al., 2018a) and under mechanically perturbed conditions (Santuz et al., 2020a), as well as on slippery ground or on a narrow beam (Martino et al., 2015). In our recent study (Janshen et al., 2020), we also identified a widening of the motor primitives in MSP, indicating an increased robustness in motor control compared with HP. This widening was present in the individual preferred speed, as well as in a reduced fixed speed. The neural strategy of motor primitive widening was also observed in sensory impaired animals (Santuz et al., 2019). From our previous work, we concluded that the neuromuscular system uses wider control signals (i.e., of longer duration) to create a temporal overlap between chronologically-adjacent synergies. Thus, the widening increased the fuzziness (Meghdadi, 2004; Gentili, 2018) of the temporal boundaries in the modular organization of walking to regulate motor function through robust control (Santuz et al., 2018a, 2020a; Janshen et al., 2020). In a recent study of HP, we observed changes in the width of motor primitives in relation to velocity during level walking and running (Santuz et al., 2020b). While increased speeds, especially in running, resulted in a widening in motor primitives, no differences were found between slow and close to preferred walking speed. Compared with HP, MSP typically demonstrate lower walking speeds (Benedetti et al., 1999; Lencioni et al., 2016; Comber et al., 2017; Janshen et al., 2020). Therefore, it remains unclear if the adjustment of the robustness in motor control of MSP might be demand-specific, e.g., depending on the mechanical demands of the movement task.

An increased demand in walking occurs in daily life when walking up ramps. Inclined walking requires larger muscle work of the hip extensors and plantar flexors in the stance phase (Silder et al., 2012) mainly to generate enough propulsion to accelerate the center of mass forward and upward. In this context, increased muscle work in the hip muscles in healthy adults has been reported (Devita et al., 2008; Wall-Scheffler et al., 2010; Alexander and Schwameder, 2016) where the increases in muscle activation were considerably higher than in the plantar flexors (Franz and Kram, 2012). Investigating the modular organization of motor control in HP during level and inclined walking, we found a similar amount of muscle synergies in the two conditions (Janshen et al., 2017; Santuz et al., 2017a). While Rozumalski et al. (2017) reported no differences in the modular organization between slopes (0, 10, and 12°) and speeds (100, 110, and 120% of the preferred speed), we observed alterations in the similarities of the motor primitives and motor modules during level and inclined walking at 10°. Besides the possible influences of the different numbers of recorded muscles, a major possible explanation for the inconsistent results could be the reduced velocity at a fixed speed of 0.7 m/s, ~50% of the participants' preferred speed (Janshen et al., 2017). We interpreted the differences in the modular organization as a mechanism to adjust the neuro-motor coordination patterns to the increased mechanical demand of inclined walking.

The purpose of this study was to investigate the modular organization of locomotion in MSP compared with age-matched HP during level and inclined walking to identify the potential demand-specific adjustments in motor control output associated with pathology-related neuromuscular impairments of MSP. We hypothesized a widening of the motor primitives in MSP to increase the overlap of temporally-adjacent muscle synergies, especially during inclined walking, as a strategy to increase the robustness of motor control and to compensate the demand- and pathology-related internal disturbances.

## Materials and Methods

The results presented in the current study are part of a research project in which the participants performed different walking conditions on a treadmill including level walking at different speeds and inclined walking (Janshen et al., 2020).

### Participants

One group of 10 female HP (age: 56 ± 4 years, body mass: 68 ± 16 kg, body height: 1.73 ± 0.07 m, body mass index (BMI): 23.0 ± 3.3 kg/m^2^) and a second group of 10 female MSP (age: 55 ± 8 years, body weight: 65 ± 9 kg, body height: 1.68 ± 0.05 m, BMI: 23.0 ± 2.5 kg/m^2^) volunteered in the experiments. The group of HP was physically active, did not use orthotic insoles, and had no known history of neurological or motor disorders or injuries over the 6 months prior to the measurements. Using a short questionnaire, six participants were identified as right leg dominant and four as left leg dominant in the HP group. The participation criteria of the study for MSP included the age of 45–70 years, the ability to walk a minimum of 500 m without assistance or assistive devices, and a common experience in treadmill walking. The patients were diagnosed for MS since 8 ± 2 years, all as relapsing remitting MS. The left leg in five patients and the right leg in the other five were more affected. At the time of measurements, they had no MS attack (i.e., worsening symptoms) within the last 3 months, and the averaged Expanded Disability Status Scale (EDSS) (Kurtzke, 1983) was 3.0 ± 1.0. For ethical reasons, patients did not lower or interrupt their continuous medication for the measurements. On a questionnaire providing a scale from 1 (never) to 5 (always), patients reported to have “rarely” (2 ± 1) difficulties in walking inside or outside their homes, respectively. Experiencing general gait or balance problems was on average classified as “occasionally” (3 ± 1). All participants of both groups had common experience in treadmill walking due to their physical activity or their rehabilitation procedures. This study was reviewed and approved by the Ethics Committee of the Humboldt-Universität zu Berlin (HU-KSBF-EK-2017003). In accordance with the Declaration of Helsinki, all participants gave written informed consent for the experimental procedure.

### Experimental Design

Prior to measurements, the preferred walking speed of each participant was obtained on a treadmill (mercury; H-p-cosmos Sports & Medical GmbH, Germany) using the method of limits (Treutwein, 1995). From an initial 0.8 m/s, the velocity was randomly increased by 0.02–0.05 m/s in varying time intervals of 5–10 s until the participant confirmed his/her preferred speed. After a short rest, the procedure was repeated. This time, the velocity was decreased, starting at an initial speed that was 0.5–1.0 m/s higher than the preferred speed. Final preferred speed was calculated by averaging both confirmed speeds. If the difference between tests was larger than 10%, the test was repeated. Thereafter, all participants randomly performed two trials (120 s each) at two walking conditions on the treadmill. The different conditions were level and inclined walking at a treadmill slope of 6%, both at 85% of the individual preferred speed. Resting time between trials was around 5 min. The measurements of the MSP were scheduled in their self-reported individual high-performance time within the day (10:15 a.m. ± 30 min to 1:30 p.m. ± 45 min). In each trial, the recordings began after a preliminary habituation phase in treadmill walking of around 30 s.

### Gait Cycle Assessment

During walking, plantar pressure distributions were measured at 120 Hz by a pressure plate (FDM-THM-S; zebris Medical GmbH, Germany) integrated in the treadmill. The pressure plate was synchronized with the electromyography (EMG) data. To identify gait cycles, the pressure data were extracted as raw data using the proprietary software (WinFDM-T v2.5.1; zebris Medical GmbH, Germany). The gait cycles were obtained using a validated custom algorithm that extracts touchdown and lift-off times and position of both feet (Santuz et al., 2016). In addition to the contact times, the pressure data were used to quantify step lengths and cadence. The data of 30 gait cycles for each of the four recorded trials per participant were averaged.

### Muscle Activity Assessment

Using pairs of Ag/AgCl electrodes (N-00-S, Ambu, Denmark) for bipolar surface EMG according to the SENIAM standards (Hermens et al., 2000), we recorded the activity of 13 ipsilateral hip and leg muscles of the dominant leg (6 × right and 4 × left leg) of the HP and the more affected leg (5 × right and 5 × left leg) of the MSP. The EMG signals were recorded at a frequency of 1,000 Hz and a resolution of 16 bit. The wireless system used (myon AG, Switzerland) had a built-in zero lag band-pass filter (5–500 Hz, 3 dB/oct, 4th order) (Santuz et al., 2017a; Janshen et al., 2020). The recorded muscles at the hip included the gluteus medius (ME), maximus (MA), and tensor fasciae latae (TF). At the upper leg, we measured the muscles rectus femoris (RF), vastus medialis (VM) and lateralis (VL), semitendinosus (ST), and the long head of the biceps femoris (BF). The lower leg muscles included the tibialis anterior (TA), peroneus longus (PL), gastrocnemius medialis (GM) and lateralis (GL), and soleus (SO). During the walking tasks, 30 consecutive gait cycles were recorded. Afterwards, signals were high-pass filtered at a cut-off frequency of 50 Hz and full-wave-rectified and low-pass filtered at a cut-off frequency of 20 Hz, both using the 4th order zero-lag IIR Butterworth filters (Santuz et al., 2017a); the EMG amplitude was normalized to the maximum activation of each muscle recorded for each trial of each condition for the respective participant (Oliveira et al., 2014; Santuz et al., 2018b; Janshen et al., 2020). Each EMG envelope was time-normalized to 200 data points per gait cycle. Each gait cycle was divided by 100 data points for the stance phase and 100 data points for the swing phase (Santuz et al., 2018b). From the rectified and filtered and normalized EMG signals, we calculated a co-contraction index (CCI) as the ratio between the averaged joint flexors and extensors at the hip, knee, and ankle joints, respectively. The ratios were calculated as follows:

(1) $$\begin{array}{l} \text{Hip}:\frac{\left(TF+RF\right)/2}{\left(ME+MA\right)/2} \end{array}$$

(2) $$\begin{array}{l} \text{Knee}:\frac{\left(BF+ST\right)/2}{\left(RF+VM+VL\right)/3} \end{array}$$

(3) $$\begin{array}{l} \text{Ankle}:\frac{TA}{\left(GM+GL+SOL\right)/3} \end{array}$$

The CCI for each joint was calculated for the stance and swing phases of each step per participant. Each of both values was averaged across all steps for each trial, then averaged across both trials of the same walking condition for each participant, and finally averaged across all participants within one group for the level and inclined walking, respectively.

### Modular Organization Assessment

Muscle synergies were extracted for each trial using a customized script (Santuz et al., 2017a) (R v3.6.3, R Core Team, 2020, R Foundation for Statistical Computing, Vienna, Austria) based on the classical Gaussian non-negative matrix factorization (NMF) algorithm (Lee and Seung, 1999, 2001). The synergy extraction procedure is described in detail in our previous studies (Santuz et al., 2018a,b). In short, the original data matrix containing 13 rows (number of muscles) and 6,000 columns (200 data points times 30 consecutive strides per trial) was factorized into two smaller matrices, representing the motor primitives (H) and the motor modules (W) for a certain number of synergies. We applied a method based on the *R*^2^ similarity between the original and reconstructed data sets obtained for each number of synergies from 1 to 10 as described in previous work of our group (Santuz et al., 2018a,b; Janshen et al., 2020). Consecutively removing lower synergy numbers, the specifically sufficient number of synergies required to reconstruct the original EMG signals of each trial was reached, when the mean squared error between the most linear part of the *R*^2^ vs. the number of synergy curve compared with a linear regression fell below 10^−5^. This approach avoided the setting of a threshold to the reconstruction quality. For further analysis, we split the concatenated motor primitives into the single gait cycles. Occasionally, synergies derived by the factorization process can be modeled from a combination of two or more simpler synergies. As stated in our previous work, we classified fundamental and combined synergies. Fundamental synergies are characterized by motor primitives demonstrating a single major activation peak (Janshen et al., 2017; Santuz et al., 2017a,b). A synergy with more than one activation peak in the motor primitives and a combination of the respective associated muscle contributions in the motor modules is characterized as a combined synergy. We used an established procedure consisting of a combination of visual inspection and automated iterative recognition based on a curve-fitting-model (Janshen et al., 2017, 2020; Santuz et al., 2017a,b; Santuz et al., 2018a). In the data, of the current study, about 20% of the total extracted synergies were classified as combined synergies. This level of occurrence was independent of participant groups and walking conditions.

### Metrics for Comparison of Curves

For each participant and each walking condition, we included both trials, each consisting of concatenated 30 consecutive strides. Thus, we accounted for the potential variations within participants across trials, increasing the information value of the measured parameters. We evaluated the similarities of the motor primitives between HP and MSP by calculating the coefficient of determinations (*R*^2^) defined as

(4) $$\begin{array}{l} R^{2}=1-\;\frac{{\left(H_{1}\;-{\;H}_{2}\right)}^{2}}{{\left(H_{1}\;-\;\bar{H_{1}}\right)}^{2}} \end{array}$$

where any two metrics of *H*_1_ and *H*_2_ of equal dimensions were compared. Separately for each of the respective motor primitives in each of the walking conditions, 10 within group similarity values for each of both groups (e.g., w HP and w MSP) were obtained by averaging the similarities of each individual motor primitive compared with the respective participant group average motor primitive (Janshen et al., 2020).

For the motor primitives, we characterized the timing and duration of the activation by evaluating the full width at half maximum (FWHM) and the center of activity (CoA), respectively. The FWHM represents the number of points exceeding half of the curve's maximum, after subtracting the minimum within the respective gait cycle (Cappellini et al., 2016; Janshen et al., 2020). As described in previous studies, the CoA is derived from the gait cycle-related position in time of the center of mass of the motor primitive (Ivanenko et al., 2006; Martino et al., 2014; Cappellini et al., 2016; Santuz et al., 2018a). We calculate the frequency of the overlapping intervals of any motor primitives for each time interval relative to gait cycle. An overlap occurred when at least two primitives were exceeding half maximum at the same time. The FWHM, CoA, and overlaps were calculated per gait cycle and then averaged for the respective participant and walking condition. For every trial, FWHM and CoA were calculated for each gait cycle and then averaged over the 30 gait cycles to proceed with the statistical analysis.

### Statistical Analysis

A linear regression model was used to evaluate the effect of the participant group (HP, MSP) including gait speed as a covariate on the respective analyzed parameters. The Shapiro-Wilk test confirmed the normal distribution of the regression residuals of all analyzed parameters. An ANOVA using the described regression model was performed to evaluate the differences between HP and MSP for both walking conditions, separately. We performed the analysis for the temporal and spatial walking parameters; CCI at the hip, knee, and ankle joints of the stance and swing phases; *R*^2^ similarities; FWHM; and CoA of the motor primitives. According to the aim of this study, we focused on the parameters of all muscle synergies that can be paired across HP and MSP (e.g., the fundamental synergies). The effect size of the statistical differences between the groups was calculated as the Hedges' g including the correction for small sample sizes (Hedges, 1981). We used a two-way ANOVA for repeated measures with factors participant group and muscle, followed by a Tukey *post-hoc* analysis with p-value adjustment (Benjamini–Hochberg) to evaluate the differences in the motor modules separately for both walking conditions. All the levels of significance were set to α = 0.05. For the statistical analyses, we used R v3.6.3.

## Results

### Gait Parameters

In MSP, preferred gait speed was significantly (*p* = 0.002, *g* = 1.10) lower (0.8 ± 0.4 m/s) than in HP (1.3 ± 0.1 m/s), and consequently, the gait velocities at 85% of the preferred speed were also significantly different (MSP = 0.7 ± 0.3 vs. HP = 1.0 ± 0.2 m/s). During level and inclined walking, we observed a significantly longer gait cycle duration in MSP than in HP. Step length was significantly shorter in MSP during level walking. The respective averages, significances, and effect sizes are given in Table 1. Compared with the HP, the MSP demonstrated significantly longer relative contact time and double stance times, whereas consequently, the relative swing times were significantly reduced. In addition, the cadence was significantly lower in MSP than in HP.

**Table 1:** Gait parameter measured by the pressure distribution (average ± standard deviation) for the healthy participants (HP) and patients with multiple sclerosis (MSP) for level and inclined walking at 85% of the preferred speed (HP = 1.0 ± 0.2 m/s, MSP = 0.7 ± 0.3).

		**Gait parameters**			
**Walking**	**Gait parameter**	**HP**	**MSP**	**p-Value**	**Hedges' g**
Level	Step length (m)	0.78 ± 0.05	0.63 ± 0.23	0.003^*^	−0.86
	Gait cycle time (s)	1.11 ± 0.06	1.29 ± 0.15	0.002^*^	1.49
	rel.ST (% gait cycle)	67 ± 3	71 ± 6	0.038^*^	0.96
	r.DST (% gait cycle)	16 ± 2	22 ± 7	0.024^*^	−1.09
	r.SWT (% gait cycle)	33 ± 3	29 ± 6	0.006^*^	0.96
	Cadence (steps/min)	107 ± 7	93 ± 14	0.010^*^	−1.22
Inclined	Step length (m)	0.78 ± 0.08	0.64 ± 0.22	0.005	−0.76
	Gait cycle time (s)	1.17 ± 0.09	1.29 ± 0.15	0.034^*^	0.93
	r.ST (% gait cycle)	67 ± 2	73 ± 5	>0.001^*^	1.31
	r.DST (% gait cycle)	17 ± 2	23 ± 6	0.001^*^	−1.20
	r.SWT (% gait cycle)	33 ± 2	27 ± 5	>0.001^*^	−1.31
	Cadence (steps/min)	103 ± 9	92 ± 13	0.030^*^	−0.95

*The stance time (r.ST), double stance time (r.DST), and swing time (r.SWT) are related to the respective gait cycle times of the individual steps*.

* *Statistically significant (p < 0.05) differences*.

### Muscle Co-contractions

In level and inclined walking, the MSP demonstrated no significantly different muscular co-contraction during the stance phase at the hip and knee joints (Table 2). In contrast, the CCI at the ankle joint during stance was significantly higher in MSP than in HP.

**Table 2:** Co-contraction indices (CCIs, average values ± standard deviations) at the hip, knee, and ankle joints in healthy participants (HP) and patients with multiple sclerosis (MSP) during level and inclined walking at 85% of the preferred speed (HP = 1.0 ± 0.2 m/s, MSP = 0.7 ± 0.3).

			**CCIs**			
**Walking**			**HP**	**MSP**	**p-Value**	**Hedges' g**
**Level**	Hip	Stance	0.81 ± 0.29	0.89 ± 0.39	0.599	0.212
		Swing	0.74 ± 0.34	0.73 ± 0.42	0.944	−0.029
	Knee	Stance	0.54 ± 0.25	0.85 ± 0.34	0.070	0.957
		Swing	1.80 ± 0.72	1.70 ± 0.80	0.794	−0.127
	Ankle	Stance	1.02 ± 0.41	1.51 ± 0.40	0.025^*^	1.153
		Swing	2.47 ± 0.83	3.33 ± 1.30	0.068	0.748
**Inclined**	Hip	Stance	0.83 ± 0.22	0.85 ± 0.23	0.859	0.079
		Swing	0.83 ± 0.35	0.67 ± 0.22	0.240	−0.518
	Knee	Stance	0.77 ± 0.35	0.91 ± 0.38	0.471	0.349
		Swing	1.86 ± 0.79	1.76 ± 0.97	0.834	−0.105
	Ankle	Stance	1.25 ± 0.23	1.66 ± 0.48	0.042^*^	1.027
		Swing	3.34 ± 1.16	3.57 ± 1.16	0.678	0.186

* *Statistically significant (p < 0.05) differences between the groups (HP/MSP)*.

### Modular Organization

**Figure 1:**
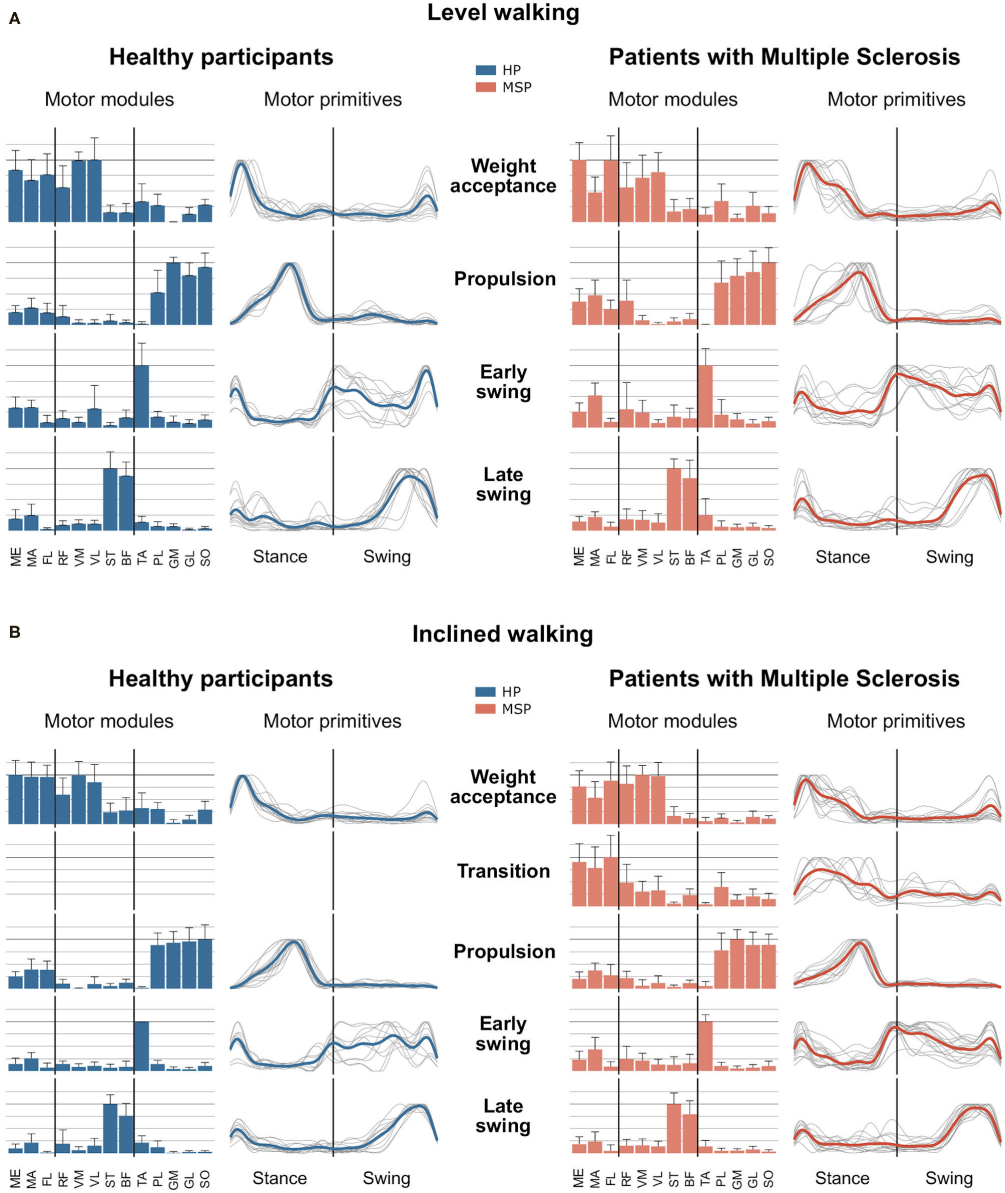
Averages and standard deviations of the motor modules and averaged and individual motor primitives of the fundamental synergies for healthy participants (HP) and patients with multiple sclerosis (MSP) during level walking **(A)** and inclined walking **(B)** at 85% of the preferred speed (HP = 1.0 ± 0.2 m/s, MSP = 0.7 ± 0.3). The motor modules are presented on a normalized y-axis base. Included muscles are named in the methods. For the motor primitives, the x-axis full scale represents one gait cycle (stance and swing normalized to the same amount of points and divided by a vertical line), and the y-axis the normalized amplitude of the muscles gluteus medius (ME), maximus (MA), tensor fasciae latae (TF), rectus femoris (RF), vastus medialis (VM), lateralis (VL), semitendinosus (ST), long head of biceps femoris (BF), tibialis anterior (TA), peroneus longus (PL), gastrocnemius medialis (GM), and lateralis (GL), and soleus (SO).

In the current study, a minimum number of 3.7 ± 0.6 synergies in HP and 3.4 ± 0.6 synergies in MSP were sufficient to reconstruct the recorded EMG activity of the lower extremity during level walking (Figure 1). The observed difference in the number of synergies between HP and MSP was not significant (*p* = 0.113, *g* = 0.80). The four synergies can be attributed to specific movement tasks within the gait cycle. In level walking, the first synergy functionally refers to the weight acceptance of the early stance phase and mainly represents the activity of the knee extensor muscles (HP). In MSP, this synergy was dominated by the hip stabilizers. The second synergy describes the propulsion phase and is dominated by the activity of the plantar flexor muscles. The third synergy identifies the early swing phase and predominantly represents the activity of the dorsiflexors. The fourth synergy occurring at the late swing involves the activity of the knee flexors, reflecting their importance in the preparation of the next step.

In contrast, during inclined walking, we observed a significantly (*p* = 0.001, *g* = 1.83) larger number of synergies in MSP (4.3 ± 0.7) than in HP (3.4 ± 0.6). In 60% of the MSP, an additional fifth synergy was necessary to sufficiently reconstruct the EMG activity. The main activation peak of this synergy was timed at 27% of the stance phase, between the synergies of the weight acceptance (12% stance phase) and propulsion (64% stance phase). The synergy was dominated by the activity of the recorded hip muscles. The fifth synergy was not demonstrated by any of the HP (Figure 1). Compared with the MSP, the HP demonstrated significantly higher within group *R*^2^ similarities of the motor primitives for the propulsion and early swing during level walking, as well as for all motor primitives during inclined walking (Table 3).

**Table 3:** Similarities of motor primitives, indicated as *R*^2^ values (average ± standard deviation) within the groups of healthy participants (w HP) and patients with multiple sclerosis (w MSP), as well as for the comparison of between the groups (Btw Groups).

		***R***^****2****^ **similarities**			
**Walking**	**Muscle synergy**	**w HP (*R*^**2**^)**	**w MSP (*R*^**2**^)**	***p***	**Hedges' g**
Level	Weight acceptance	0.465 ± 0.172	0.486 ± 0.063	0.726	0.157
	Propulsion	0.800 ± 0.042	0.403 ± 0.117	<0.001^*^	−4.330
	Early swing	0.339 ± 0.298	-0.470 ± 0.352	<0.001^*^	−2.373
	Late swing	0.428 ± 0.162	0.432 ± 0.134	0.946	−0.030
Inclined	Weight acceptance	0.693 ± 0.069	0.196 ± 0.103	<0.001^*^	−5.445
	Propulsion	0.733 ± 0.057	0.578 ± 0.086	<0.001^*^	−2.040
	Early swing	0.166 ± 0.384	-0.314 ± 0.281	0.006^*^	−1.365
	Late swing	0.695 ± 0.098	0.456 ± 0.054	<0.001^*^	−2.901

*R^2^ values are given for level and inclined walking at 85% of the preferred speed (HP = 1.0 ± 0.2 m/s, MSP = 0.7 ± 0.3)*.

* *Statistically significant (p < 0.05) differences between the groups (HP/MSP)*.

**Figure 2:**
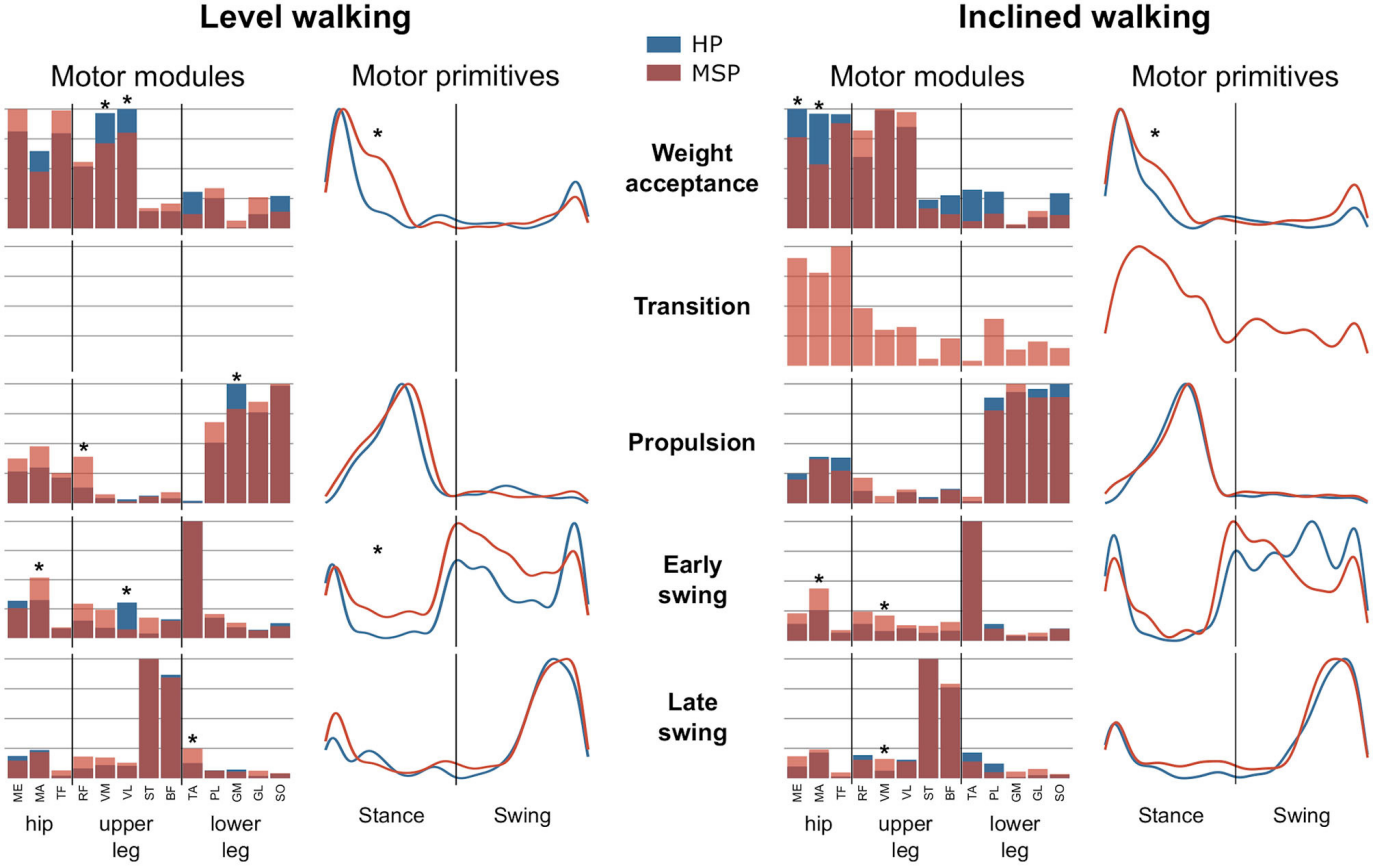
Average motor modules and motor primitives of the fundamental synergies for level and inclined walking. The motor modules are presented on a normalized y-axis base. Included muscles at the hip were gluteus medius (ME), maximus (MA), and tensor fasciae latae (TF); at the thigh rectus femoris (RF), vastus medialis (VM) and lateralis (VL), semitendinosus (ST), and the long head of biceps femoris (BF). Lower leg muscles were tibialis anterior (TA), peroneus longus (PL), gastrocnemius medialis (GM) and lateralis (GL), and soleus (SO). For the motor primitives, the x-axis full scale represents one gait cycle (stance and swing normalized to the same amount of points and divided by a vertical line), and the y-axis the normalized amplitude. Asterisks denote significant differences (*p* < 0.05) between healthy participants (HP) and patients with multiple sclerosis (MSP) for *post-hoc* tests in the motor modules and significant differences in the full width at half maximum between the groups.

During level walking, the MSP showed significantly (*p* < 0.001 and 0.016, *g* = 1.65 and 1.16) larger FWHM values at the weight acceptance (32.2 ± 8.1%) and early swing motor primitives (75.0 ± 24.9%) than the HP (21.5 ± 4.0 and 49.0 ± 16.1, respectively). During inclined walking, the FWHM in the motor primitives of the weight acceptance synergy in the MSP (30.7 ± 12.1%) was significantly (*p* = 0.001, *g* = 1.23) increased compared with the HP group (18.9 ± 4.5%) (Figure 2).

In MSP, the CoA in level walking was significantly delayed in the weight acceptance motor primitive, whereas it occurred significantly earlier in the early swing motor primitive compared with HP. During inclined walking, the motor primitive of the late swing synergy was significantly delayed in the MSP compared with the HP group (Table 4).

**Table 4:** Center of activity (CoA, average values ± standard deviations) of the motor primitives for the healthy participants (HP) and patients with multiple sclerosis (MSP) for level and inclined walking at 85% of the preferred speed (HP = 1.0 ± 0.2 m/s, MSP = 0.7 ± 0.3).

		**CoA**			
**Walking**	**Muscle synergies**	**HP (%)**	**MSP (%)**	***p***	**Hedges' g**
Level	Weight acceptance	14.3 ± 4.3	20.1 ± 4.6	0.011^*^	1.255
	Propulsion	53.0 ± 2.1	52.7 ± 7.6	0.881	−0.051
	Early swing	67.6 ± 8.9	46.3 ± 16.1	0.002^*^	−1.578
	Late swing	78.4 ± 7.1	80.8 ± 5.4	0.396	0.361
Inclined	Weight acceptance	17.5 ± 4.4	18.0 ± 8.0	0.883	0.062
	Transition		37.3 ± 14.4		
	Propulsion	56.2 ± 3.9	55.7 ± 6.9	0.836	−0.085
	Early swing	60.5 ± 12.7	37.7 ± 19.4	0.004^*^	−1.327
	Late swing	89.5 ± 0.9	89.3 ± 1.9	0.779	−0.125

*Values are given in % stance phase for the weight acceptance, transition, and propulsion motor primitives and in % swing phase for the early and late swing motor primitives*.

* *Statistically significant (p < 0.05) differences between the groups (HP/MSP)*.

**Figure 3:**
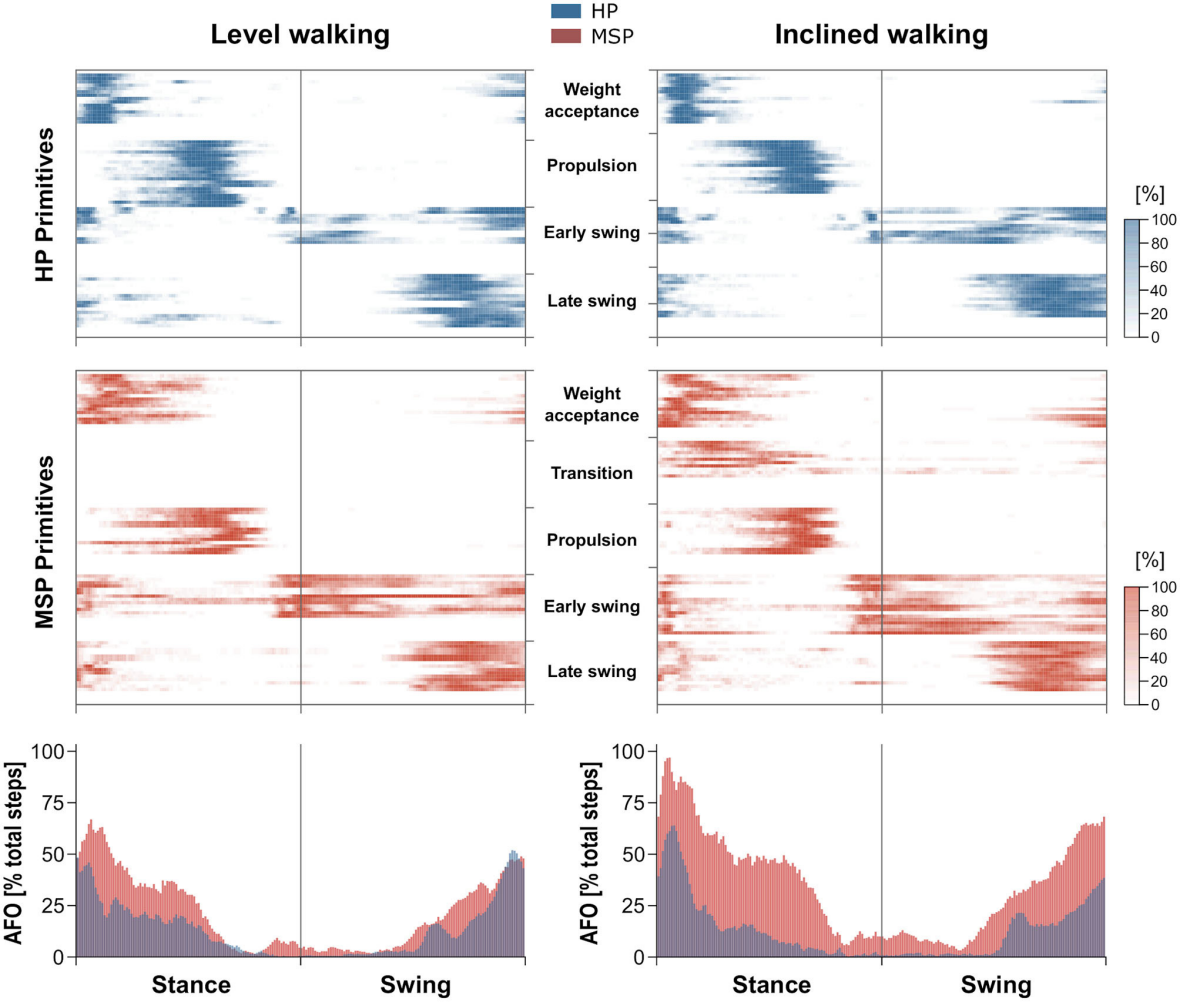
Overlapping time intervals of motor primitives during level walking (left) and inclined walking (right) both at 85% of the preferred speed (HP = 1.0 ± 0.2 m/s, MSP = 0.7 ± 0.3). The heat maps show the individual motor primitives when exceeding half maximum activation for healthy participants (HP, blue) and multiple sclerosis patients (MSP, red). Darker colors indicate more exceedances across all 30 gait cycles per participant. At the bottom, the averaged frequency of overlaps (AFO) across all gait cycles and all participants per group are shown. For all graphs, the x-axis full scale represents one gait cycle (stance and swing normalized to the same amount of points).

For both walking conditions, overlapping motor primitives occurred in the weight acceptance and early push-off, as well as in the late swing of the gait cycle, with the highest number of overlaps during or shortly after touchdown. During level walking, the MSP showed a larger number of overlaps than the HP. These occurred at the weight acceptance and push-off motor primitives, as well as at the early and late swing motor primitives. Thus, the averaged frequencies of overlaps in the respective time phases of the gait cycle for MSP were increased compared with HP (Figure 3).

During inclined walking, the higher averaged frequencies of overlaps in MSP compared with HP were more obvious than in level walking (Figure 3). In the stance phase, this could mainly be attributed to the overlap of the motor primitives of the weight acceptance and the additional synergy of the transition and the propulsion synergy. In the swing phase, the increased overlaps mainly resulted from the motor primitives of the early and late swing synergies.

During level walking, the weight acceptance synergy in MSP is dominated by the hip extensor (MA) and hip stabilizer (TF), whereas the contributions of the knee extensors (VM, *p* = 0.005, *g* = −1.05; VL, *p* = 0.017, *g* = −0.80) were significantly reduced compared with HP. In the propulsion synergy, we identified significantly higher relative contributions of the RF (*p* = 0.005, *g* = 1.37) and a reduction of the GM (*p* < 0.001, *g* = −1.30) in MSP compared with HP. In the early swing synergy, the relative contribution of the MA muscle in MSP was significantly (*p* = 0.003, *g* = 1.90) higher, whereas the one of the VL muscle was significantly (*p* = 0.002, *g* = −1.07) lower than those in HP. In addition, the contribution of the TA (*p* = 0.03, *g* = 0.86) to the late swing synergy in MSP was significantly increased compared with HP. During inclined walking, MSP demonstrated significant reductions of the relative contributions for the ME (*p* = 0.03, *g* = −0.72) and MA (*p* < 0.001, *g* = −1.33) muscles in the weight acceptance synergy (Figure 2). In contrast, the muscles MA and VL demonstrated significantly (*p* < 0.001, *g* = 1.17, and *p* = 0.003, *g* = 1.18, respectively) higher relative contributions to the early swing synergy in MSP than in HP. The same was found for the VM muscle (*p* = 0.02, *g* = 1.34) in the late swing synergy. In both walking conditions, walking speed had no statistically significant influence on any of the observed differences between HP and MSP.

## Discussion

In the current study, we compared patients with MS and age-matched HP in two walking conditions (i.e., level and inclined walking) to investigate the modular organization and potential demand-specific adjustments in motor control. Compared with HP, we observed a widening in the motor primitives (the basic activation patterns) in MSP in both walking tasks. The widening was not related to co-contractions that were increased only at the ankle joint in MSP compared with HP. The widening promoted greater overlaps of the temporal components of muscle synergies in MSP. The found overlapping of the motor primitives increased from level to inclined walking in MSP, indicating a demand-specific modulation of motor control in this group. Moreover, during inclined walking, the MSP demonstrated an additional muscle synergy mainly representing the hip muscle activation. Our findings show that MSP modified their modular organization during locomotion in order to compensate for pathology-related deficits and to ensure robust motor control. Notably, our results revealed that the modulation of motor control depends on task demand in MSP.

Consistent with other literature reports and our previous work, four muscle synergies were sufficient to describe level walking in HP (Ivanenko et al., 2004; Cappellini et al., 2006; Oliveira et al., 2014; Martino et al., 2015; Janshen et al., 2017; Santuz et al., 2018a) and MSP (Lencioni et al., 2016; Janshen et al., 2020). In this study, the MSP were characterized by mild to moderate motor impairment. We reasoned that MSP with a higher level of impairment might demonstrate changes in the number of muscle synergies even during level walking (Clark et al., 2010; Allen and Neptune, 2012; Cheung et al., 2012). We introduced the inclined walking condition to increase the demand within the overall pattern of locomotion for both participant groups. Inclined walking requires larger muscle work of the hip extensors and plantar flexors in the stance phase (Silder et al., 2012; Janshen et al., 2017) mainly to generate enough propulsion to accelerate the center of mass forward and upward. In healthy adults, increased muscle work in the hip muscles has been reported (Devita et al., 2008; Wall-Scheffler et al., 2010; Alexander and Schwameder, 2016) where the increases in muscle activation were considerably higher than in the plantar flexors (Franz and Kram, 2012).

During inclined walking, the age-matched older adults of the HP group maintained the overall motor control pattern of four muscle synergies as we also reported for the lower extremity during inclined walking in younger adults (Janshen et al., 2017). An increased activation in the hip muscles was represented by increased relative contributions of the respective muscles in the motor modules of the weight acceptance synergy. These findings are also consistent with a modeling study (McGowan et al., 2010) reporting an effect of additional mass and weight on the motor modules but not necessarily on the timing of motor primitives in level walking. In contrast, the MSP demonstrated an additional fifth synergy during inclined walking that was dominated by the hip muscles. We argue that the additional synergy in MSP reveals a demand-specific adjustment of the modular organization in neuromuscular control. The extra synergy occurred in addition to a widening of the motor primitive of the weight acceptance and early swing synergy in MSP compared with HP. At the same time, MSP compared with HP did not show larger co-contractions at the hip or knee joint during level or inclined walking. Compared with HP, MSP only demonstrated an increased co-contraction at the ankle joint in the stance phase during inclined walking. These results indicate a negligible association between the observed widening of the weight acceptance and early swing motor primitives and increased ankle joint co-contraction, as the lower leg muscles show only minor contributions to the named synergies. From our previous work, we concluded that the neuromuscular systems create a temporal overlap of chronologically-adjacent synergies using wider control signals (i.e., longer duration of the main activation patterns). Thus, the increased fuzziness (Meghdadi, 2004; Gentili, 2018) of the temporal boundaries in the modular organization of walking helps to regulate motor function through robust control (Santuz et al., 2018a, 2020a; Janshen et al., 2020). From this perspective, the extra muscle synergy in MSP most likely was necessary to provide sufficient robustness of the neuromuscular control under increased demand resulting from the uphill inclination of the treadmill.

The extra synergy further increased the overlap between the chronologically-adjacent synergies, facilitating the transition between temporal boundaries through higher fuzziness of the modular organization. This provides evidence that demanding locomotor conditions require a greater modulation in motor control in MSP to ensure a safe performance of the movement task. The greater modulation of motor control in MSP was initiated by the need to generate enough propulsion during the stance phase while coping with individual motor impairments. These impairments include reduced motor capacities of the leg muscles (Lambert et al., 2001; Thoumie et al., 2005; Kalron et al., 2011), as well as reduced proprioceptive feedback (Fling et al., 2014). The reduced muscle strength has been shown for MSP with an EDSS of 3.5, a score comparable to the current study (Lambert et al., 2001), for more severely affected patients with an EDSS of up to 6.5 (Thoumie et al., 2005), as well as for patients at an early stage of the disease and only mild symptoms at an EDSS of 1.7 (Kalron et al., 2011). The impairments were associated with similar differences in the gait spatiotemporal parameters as presented in the current study. The reduced proprioceptive feedback in MSP has been attributed to neural deficits (Fling et al., 2014) in the spinal (Cameron et al., 2008) and supraspinal (Wylezinska et al., 2003) domains attributed to the MS disease.

Therefore, the increased robustness and fuzziness of the motor control system based on wider and more overlapping motor primitives in MSP than in HP may be attributed to both a) the compensation of pathology-induced reductions in mechanical output capacities of specific muscles and b) the compensation of a potentially reduced sensory (e.g., proprioceptive) feedback. In the mouse, it has been shown that the lack of feedback from proprioceptors induces the same modulation strategy (i.e., increased width and fuzziness of motor primitives) that we found in MSP (Santuz et al., 2019). It can be assumed that the inclined walking task has been relatively more demanding for the MSP than for the HP. We propose that this demand may have required an increased robustness in motor control during the one-leg stance and particularly the push-off phase. In MSP, the activation of the hip muscles was shifted later in time toward the push-off phase. This resulted in the identified extra synergy instead of simply changing the relative contributions of the respective muscles in the motor modules within the weight acceptance synergy, as demonstrated by HP. We also found lower similarities of the respective motor primitives within the MSP group during level and especially during inclined walking than the HP, indicating higher individual variations of the overall motor control strategies. This reveals a less consistent temporal structure of muscle synergies in MSP. The large variations in the individual motor primitives of the extra synergy can be related to the individual compensation strategies to accomplish the inclined walking task. A context-related (e.g., demand-specific) modification of the motor control pattern was also reported in HP in perturbed balance tasks with different demands. During perturbations while standing on both legs in a normal, wider, or narrower position, no additional synergy was found. In contrast when standing on one leg or crouching, an extra synergy to maintain balance was observed (Torres-Oviedo and Ting, 2010). In side stepping, an additional synergy of the leg muscles was observed, when the task was performed on an unstable surface compared with stable ground (Munoz-Martel et al., 2019).

When interpreting the results, some limitations of this study should be taken into account. On the one hand, the relatively small number of exclusively female participants with relapsing remitting MS only allows for a limited generalization of the results to MSP. We recorded the muscles of only one leg, although walking is a bipedal movement that also involves the trunk. Comparing the more affected leg of MSP with the dominant leg of HP might overestimate the differences. However, for the HP, the current results closely correspond to our previous work (Janshen et al., 2017; Santuz et al., 2017a, 2020a) and to other existing literature reports (Cappellini et al., 2006; Ivanenko et al., 2008). This is also true for the MSP results during level walking (Janshen et al., 2020). In addition, the current results were generated only during treadmill walking at a preferred and a fixed speed. There might be slightly differences between treadmill and overground walking; however, muscle synergies have been shown to be generally consistent for both environments (Oliveira et al., 2016; Mileti et al., 2020).

In summary, patients with MS demonstrated a widening of the motor primitives to enhance robustness of motor control during level and inclined walking compared with HP. Moreover, the inclined walking revealed a demand-specific adjustment in the modular organization in MSP, resulting in an extra synergy and a widening of motor primitives compared with HP. This further amplified the overlap of temporally-adjacent muscle synergies to provide sufficient robustness in motor control to accomplish the more demanding motor task. These adaptations of the motor system were most likely necessary to cope with pathology-related impairments associated with deficits in the mechanical capacity of specific muscles and potentially reduced sensory feedback in MSP.

## Data Availability Statement

The collected data can be made available from the corresponding author upon request: lars.janshen@hu-berlin.de.

## Ethics Statement

The studies involving human participants were reviewed and approved by Ethikkommission der Kultur-, Sozial- und Bildungswissenschaftlichen Fakultät der Humboldt-Universität zu Berlin. The patients/participants provided their written informed consent to participate in this study.

## Author Contributions

LJ and AA: conceptualization and writing—original draft. LJ, AS, and AA: methodology, formal analysis, writing—review, and editing. LJ and AS: investigation and visualization.

## Conflict of Interest

The authors declare that the research was conducted in the absence of any commercial or financial relationships that could be construed as a potential conflict of interest.
